# Supplementary material for: COZOID: contact zone identifier for visual analysis of protein-protein interactions
Source: BMC Bioinformatics. 2018 Apr 6;19:125. doi: 10.1186/s12859-018-2113-6 (PMC5889581; doi:10.1186/s12859-018-2113-6)
Supplement: Supplementary file 2 — Software build. Executable binary file of the software tool. (ZIP 118,784 kb) [file 12859_2018_2113_MOESM2_ESM.zip › cozoid/platform/docs/testng-6.5.1-javadoc.zip!/org/testng/annotations/ITestOrConfiguration.html]

ITestOrConfiguration (TestNG)


---


|  |  |  |
| --- | --- | --- |
| **Overview** \| **Package** \| **Class** \| **Tree** \| **Deprecated** \| **Index** \| **Help** | |  |
| **PREV CLASS**   **NEXT CLASS** | **FRAMES**    **NO FRAMES**     **All Classes** |  |
| SUMMARY: NESTED \| FIELD \| CONSTR \| METHOD | DETAIL: FIELD \| CONSTR \| METHOD |  |


---


## org.testng.annotations Interface ITestOrConfiguration

**All Superinterfaces:**: IAnnotation, IParameterizable

**All Known Subinterfaces:**: IConfigurationAnnotation, ITestAnnotation

---

``` public interface ITestOrConfiguration extends IParameterizable ```

This interface captures methods common to @Test and @Configuration
Created on Dec 20, 2005

**Author:**
:   Cedric Beust

---

| **Method Summary** | |
| --- | --- |
| `java.lang.String[]` | `getDependsOnGroups()`             The list of groups this method depends on. |
| `java.lang.String[]` | `getDependsOnMethods()`             The list of methods this method depends on. |
| `java.lang.String` | `getDescription()`             The description for this method, which will be shown in the reports. |
| `java.lang.String[]` | `getGroups()`             The list of groups this class/method belongs to. |
| `long` | `getTimeOut()`             Returns the maximum number of milliseconds this test should take. |
| `void` | `setDependsOnGroups(java.lang.String[] groups)` |
| `void` | `setDependsOnMethods(java.lang.String[] dependsOnMethods)` |
| `void` | `setDescription(java.lang.String description)` |
| `void` | `setGroups(java.lang.String[] groups)` |
| `void` | `setTimeOut(long l)` |

**Methods inherited from interface org.testng.annotations.IParameterizable**

`getEnabled, getParameters, setEnabled`

**Method Detail**

### getTimeOut

```
long getTimeOut()
```

:   Returns the maximum number of milliseconds this test should take.
    If it hasn't returned after this time, it will be marked as a FAIL.

    :   **Returns:**: the maximum number of milliseconds this test should take.

---


### setTimeOut

```
void setTimeOut(long l)
```

---


### getGroups

```
java.lang.String[] getGroups()
```

:   The list of groups this class/method belongs to.

---


### setGroups

```
void setGroups(java.lang.String[] groups)
```

---


### getDependsOnGroups

```
java.lang.String[] getDependsOnGroups()
```

:   The list of groups this method depends on. Every method
    member of one of these groups is guaranteed to have been
    invoked before this method. Furthermore, if any of these
    methods was not a SUCCESS, this test method will not be
    run and will be flagged as a SKIP.

---


### setDependsOnGroups

```
void setDependsOnGroups(java.lang.String[] groups)
```

---


### getDependsOnMethods

```
java.lang.String[] getDependsOnMethods()
```

:   The list of methods this method depends on. There is no guarantee
    on the order on which the methods depended upon will be run, but you
    are guaranteed that all these methods will be run before the test method
    that contains this annotation is run. Furthermore, if any of these
    methods was not a SUCCESS, this test method will not be
    run and will be flagged as a SKIP.
    If some of these methods have been overloaded, all the overloaded
    versions will be run.

---


### setDependsOnMethods

```
void setDependsOnMethods(java.lang.String[] dependsOnMethods)
```

---


### getDescription

```
java.lang.String getDescription()
```

:   The description for this method, which will be shown in the reports.

---


### setDescription

```
void setDescription(java.lang.String description)
```


---


|  |  |  |
| --- | --- | --- |
| **Overview** \| **Package** \| **Class** \| **Tree** \| **Deprecated** \| **Index** \| **Help** | |  |
| **PREV CLASS**   **NEXT CLASS** | **FRAMES**    **NO FRAMES**     **All Classes** |  |
| SUMMARY: NESTED \| FIELD \| CONSTR \| METHOD | DETAIL: FIELD \| CONSTR \| METHOD |  |


---
